# Linking genomic and epidemiologic information to advance the study of COVID-19

**DOI:** 10.1038/s41597-022-01237-1

**Published:** 2022-03-30

**Authors:** Yiwei Wang, Jiaxin Yang, Xinhao Zhuang, Yunchao Ling, Ruifang Cao, Qingwei Xu, Peng Wang, Ping Xu, Guoqing Zhang

**Affiliations:** 1 Shanghai Information Center for Life Sciences, Shanghai Institute of Nutrition and Health, University of Chinese Academy of Sciences, Chinese Academy of Sciences, Shanghai, 200031 China; 2 National Genomics Data Center, CAS Key Laboratory of Computational Biology, Bio-Med Big Data Center, Shanghai Institute of Nutrition and Health, University of Chinese Academy of Sciences, Chinese Academy of Sciences, Shanghai, 200031 China; 3 School of Life Science and Technology, ShanghaiTech University, Shanghai, 200031 China; 4 College of Computer, Hubei University of Education, #129 Second Gaoxin Road, East Lake Hi-Tech Zone, Wuhan, 430205 China

**Keywords:** Computational biology and bioinformatics, Biotechnology

## Abstract

The outbreak of Coronavirus Disease 2019 (COVID-19) at the end of 2019 turned into a global pandemic. To help analyze the spread and evolution of the virus, we collated and analyzed data related to the viral genome, sequence variations, and locations in temporal and spatial distribution from GISAID. Information from the Wikipedia web page and published research papers were categorized and mined to extract epidemiological data, which was then integrated with the public dataset. Genomic and epidemiological data were matched with public information, and the data quality was verified by manual curation. Finally, an online database centered on virus genomic information and epidemiological data can be freely accessible at https://www.biosino.org/kgcov/, which is helpful to identify relevant knowledge and devising epidemic prevention and control policies in collaboration with disease control personnel.

## Abstract (summary)

**Table:** 

Measurement(s)	Viral Epidemiology • genetic sequence variation analysis
Technology Type(s)	digital curation • Bioinformatics
Sample Characteristic - Organism	Severe acute respiratory syndrome coronavirus 2

## Background & Summary

Severe acute respiratory syndrome coronavirus 2 (SARS-CoV-2) infected 83,088,086 people worldwide from December 2019 to December 2020, and caused 1,911,736 deaths in more than 200 countries or regions^1^. During the pandemic^1^, large amounts of COVID-19-related data and information were released from different sources. Tens of thousands of COVID-19-related research articles have been published, covering epidemiology^2–4^, clinical studies^5,6^, molecular mechanisms^7–9^, pharmaceuticals^10,11^, and vaccine research^12–15^. Literature databases are one of major information sources, and biological databases have also contributed significantly to the data pool. Different kinds of sequence data are available, including raw sequencing data, virus genome sequences, and protein sequences. Furthermore, COVID-19 data can be found in epidemiological news and reports, produced and disseminated by the World Health Organization, public health authorities, Wikipedia, and other websites^16,17^. These data have addressed COVID-19 and the SARS-CoV-2 viruses from different perspectives, but it was difficult to obtain a comprehensive understanding of COVID-19 and SARS-CoV-2 virus from these data. To take full advantage of this diversity of information, it is necessary to develop a framework to integrate data from diverse sources and heterogeneous models. With viral genomic and epidemiological data, we conducted unified structured and standardized processing and produced a new genomic epidemiology knowledge graph dataset.

## Methods

### Epidemiological data processing

The main unstructured text materials of epidemiological data were Wikipedia web pages and the published literatures. Wikipedia collated a series of reports which reviewed early confirmed cases in each country on a country-by-country scale during the early outbreak of COVID-19. By April 19, 2020, COVID-19 outbreaks had been reported in 168 countries. The epidemiological literatures were another source of unstructured text material, containing de-identified transmission and infection details, and can be considered more reliable information than Wikipedia. There were tens of thousands of COVID-19 related articles before June of 2020, but few articles were about epidemiological research. These articles were reviewed, and 33 articles were collated in which the early outbreak with COVID-19 cases information were described in detail, the bibliographic information of 33 articles was listed on https://github.com/BioMedBigDataCenter/KGCoV/blob/main/data/curated_articles.csv. Because there may be several infected individuals in one webpage or article, every person was assigned a unique case id, which may be infected or potentially infected or contacted with infected people. The information of COVID-19 cases was extracted from unstructured text materials, which was performed in a double-blinded manner by two junior curators and rechecked by another senior curator. The junior curator found out case information (report date, location, gender, age), contact history, travel history, clinical symptom (patient status, clinical symptoms, onset date), and other information. All case information was extracted by two junior curators in parallel. If the result of two curator were same, this case information was qualified. If the results were different, the senior curator would re-curate and retain qualified information directly. As some information may be lost or corrupted in the structuration procedure, the original text was kept in the “description” field and thus can be traced by the following matching step.

The main structured epidemiological data came from Xu *et al*.^16^, who collected and collated epidemiological data from multiple sources such as government reports and news. We had continuously updated Xu *et al*.’s data until November 6, 2020, and found most of the data is before June 2020. All structured data were integrated with unstructured information, so both types of data were organized with one data model to record case entries. The fields included report date, gender, age, country, location, contact information, travel history, clinical symptoms, description, and information source. The data model was compatible with Xu *et al*.’s data model, and all COVID-19 cases were structured and characterized using the unified model.

After data acquisition and structuring, quality control was focused on a few data fields, such as location, report date, age and gender. All values of location field were standardized by the Google Place API, which built a controlled vocabulary of country names based on ISO 3166, and can be as accurate as possible, including country, province or state, city, and lower levels. The values of date field were transformed to standard date format. The values of age field were adjusted as numeral values or ranges. The generic descriptions of age, such as “adult” or “child” were ignored. The values of gender field were unified as “male” or “female”.

### Genomic data processing

The main genomic data were obtained from GISAID’s EpiCoV thematic database (https://www.gisaid.org/). All involved genomes were de-duplicated according to the genome sequence, submission date, gender, patient age, submitting lab and originating lab. Sample and host information together with genome sequencing records were extracted, and were considered genome-related epidemiological data. The function data of the SARS-CoV-2 protein domains were obtained from UniProt^18^.

The raw genome sequences were filtered using BLAT (v. 36 × 5)^19^, and genomes with greater than 95% similarity to the reference genome NC_045512.2 were retained for further analysis. The whole genome variations and amino acid variations of these genomes were annotated by the multiple sequence alignment tool MAFFT(v 7.453)^20^ and ANNOVAR (2019 Oct 24 version)^21^. As nonsynonymous mutations lead to changes in the amino acid sequences of proteins, which may lead to changes in the infectivity and lethality of the novel coronavirus^22,23^, the amino acid variations were aligned to protein domains based on their position (offset on locus). In this way, genomes, proteins, and functional domain information were linked with genomic variation annotations and amino acid variations. The data sources were shown in Table 1.

**Table 1:** Data sources.

Data source	Data type	Data Format	Recorded version	Provider	URL
Wikipedia	Epidemiology	text	19 Apr. 2020	—	https://en.wikipedia.org/wiki/COVID-19_pandemic
Xu *et al*.’s data set	Epidemiology	csv	16 Nov. 2020	—	https://github.com/beoutbreakprepared/nCoV2019/
PubMed SARS-CoV-2 Literature	Epidemiology	text	Jul. 2020	National Institutes of Health	https://pubmed.ncbi.nlm.nih.gov/?term=covid-19
GISAID	Genomic	fasta	Nov. 2020	Max Planck Institute for Informatics	https://gisaid.org/CoV2020
UniProt	Viral domain information	csv	Nov. 2020	UniProt	https://covid-19.uniprot.org/uniprotkb?query=*

### Data matching

**Fig. 1:**
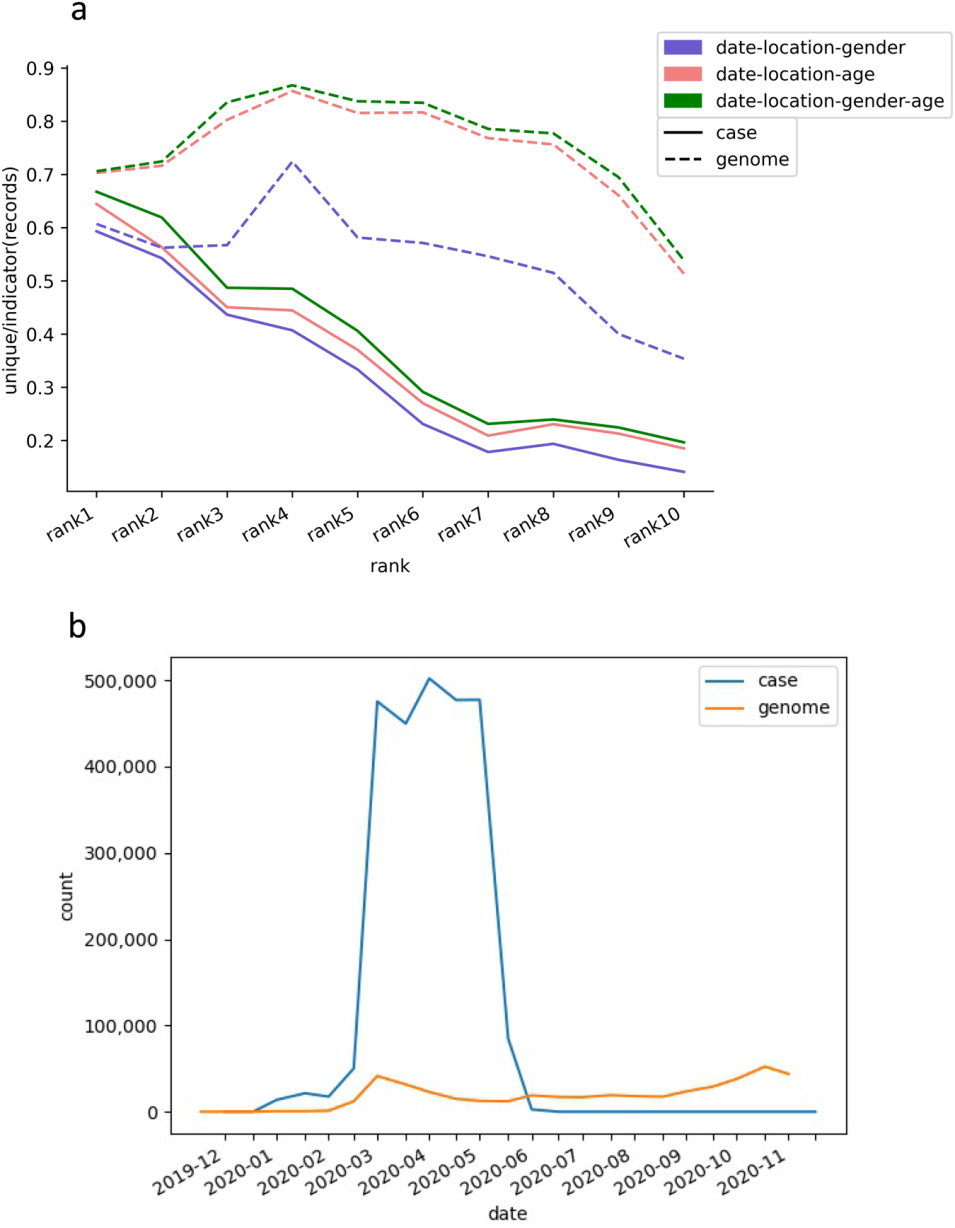
Distribution of data rank and date. (**a**) The ratio distribution of unique entries in top 10 rank case or genome data. The ratio of unique case or genome entries to all entries, was calculated by three kinds of combination of 4 indicators. (**b**) The temporal distribution of the number of cases or genomes. Blue represents the cases and orange represents the genomes. Note that because cases are multiple sources, the number of cases does not represent the actual number of reported cases, which is higher than the actual number of cases.

Once the epidemiological data and genomic information were structured and standardized from heterogeneous sources, and the both type data were matched using anonymized information of infected persons. In this study, four indicators were used as matching criteria: date, location, gender and age. The date of epidemiological data was report date, and the date of genomic data was collection date. The location values were structured as lower level of administrative division as possible, which may be from country to province/state to city. Comparatively, the combination of the four indicators was reasonable to determine the individual case or genome in the outbreak data of early phase, thus can be used to link both data. In order to evaluate the sufficiency and effectiveness of the matching criteria, the percentages of unique values were calculated in all case/genome records with three indicators (date-location-gender or date-location-age) or four indicators(date-location-gender-age), the results (Fig. 1a) shown the percentage of 4 indicators was higher than 3 indicators in genome dataset and case dataset.The temporal distribution of case reported date and genome collection date were counted every fortnight (Fig. 1b). The case number rapidly increased between March and June of 2020, some countries’ distribution also provides (https://github.com/BioMedBigDataCenter/KGCoV/blob/main/data/descriptive_statistics.xlsx), which brought great difficulties to data matching and case collection.

**Fig. 2:**
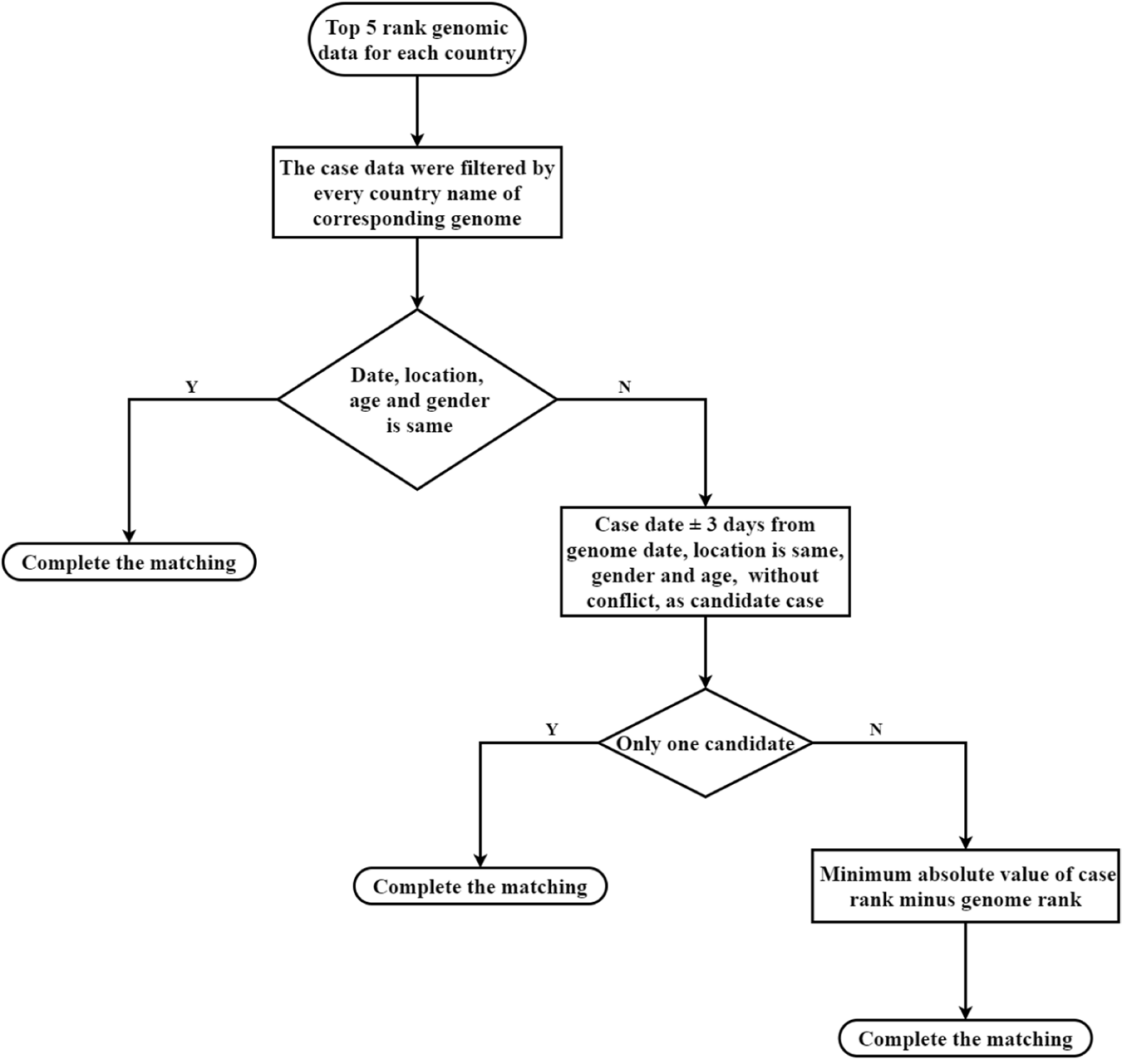
Manual curation flow chart.

The matching process include two steps as shown in Fig. 2, the initial qualified matching dataset was collating by manual curation, and the characteristic of 4 indicators were used to design and develop the in-house inferred scripts.

#### Manual curation

The early epidemiological reports were often more detailed and less ambiguous than later reports, and the first cases in an area had higher exposure and were often well studied. Therefore, cases were grouped by countries and sorted by ascending date order. Two indicators were used to identify earlier cases: “rank” and “percentile”. The rank and percentile were considered when matching genomes and cases. Firstly, the top 5 rank genome in every country were extracted from genomic data, and the case data were filtered by every country name of the corresponding genome. Secondly, four indicators in the selected genome data of each country, as mentioned above, were applied to match the selected case data. If the value of date, location, gender and age of in case dataset was the same as in the genome dataset, the case was considered to match the genome directly. Otherwise, if the report date of some cases were in the interval of ± 3 days of the collection date of genomes, the location was same, and the gender and age do not conflict (that is, the value of these fields was the same in case and genome data, and cannot be empty at the same time), the case was considered to potential match the genome. The number of potential matched case is further considered, if there was only one case data with the genome, the matching was successful. If there were multiple cases, the final matching data was determined according to the minimum absolute value of case rank minus genome rank (Fig. 2). With the elaborate governance, 285 genome-case pairs were deduced, and also used manual curation when verifying the accuracy of code matching. In this manual curated data set, the collection date and report date of most matched data were the same day (107/285, 37.54%) or one day apart (112/285, 39.30%).

#### Inference

The manual curated dataset was mostly extracted from top 5 rank genome-related epidemiological data. All potential matching pair were imputed from whole genome-related and case related epidemiological data by scripts. The inferred criterion is location is same, date is the same day or one day apart, gender and age do not conflict in both data.

The case and genome were assumed to be one matched genome–case pair cluster, containing both epidemiological and genomic information about a single patient. If there was one genome and one case in a cluster, it was very likely that the genome was sampled from the case in the cluster. If there was more than one case, it was difficult to identify a specific case in the cluster, and more detailed information was needed, such as contact, travel, genome variation, and virus typing information. In this case we can only provide information about cases that the genome might correspond to.

## Data Records

The dataset can be accessed via 10.11922/sciencedb.00818^24^ or our web platform at https://www.biosino.org/kgcov/data. Four data files are available: epidemiological data (case.tsv), genomic data (genome.tsv), genomic variation data (variant.tsv), and genomic–case pair data (case_genome.tsv). Each epidemiological and genomic record was assigned a unique id as per Research Data Alliance (RDA) guidelines^25^. The detailed description of the fields in data files is as follows:

There are some shared fields.

**gender/qc_gender:** Gender or after quality control gender. It was recorded as N/A when it was not reported.

**age/qc_age:** Age or after quality control age. It was recorded as N/A when it was not reported.

**qc_province:** Province information structured by Google API.

**country/qc_country:** Country or based on iso3166 controlled country. **location/qc_location:** Where the case or genome was collected or after structured by Google API.

**qc_continent**: The continent to which the country belongs after quality control.

**count:** The total number of confirmed cases in each country during the data collection period.

**rank:** Each country’s case or genome sample is ranked by reported_date or collection_date.

**percentile:** The ratio of the rank of each case or genome rank in each country to the total number of cases or genomes.

Unique fields in case.tsv:

**case_id:** Unique coding ID for epidemiological data.

**case_reported_date/qc_case_reported_date**: Date of case report or date after quality control, format YYYY.MM.DD.

**data_source:** Data source for the case.

**description**: The original description of the case.

**URL:** The URL of the original descriptions, which were used to validate the data.

**contactinfo**: Case exposure history, the case_id of the contact object.

**travelinfo**: Case travel history, data format as mentioned above, in order of travel date, from_location, transport, to_location.

**clinicalinfo**: The clinical symptoms of the case.

Unique fields in genome.tsv:

**virus_id**: The unique coding ID of the genome.

**genome_id**: The ID of the genome in the source database.

**virus_name**: Genome name in the source database.

**sample_collection_date/qc_sample_collection_date:** Date of sample collection or sample collection date after quality control: format YYYY.MM.DD.

**data_source:** Data source for the genome.

**specimen_source**: Sample collection method, e.g., pharynx swabs, nasopharyngeal swabs.

**Author**: The name of the submitter who submitted the genome.

**submitter_lab_address**: The address of the lab from which the sample was obtained.

**Address**: The address of the submitter who submitted the genome.

**origin_lab**: The laboratory where the sample was taken.

**origin_lab_address**: The address of the sampling lab.

Fields in variant.tsv:

**variant_id:** The location of the variation, named after the location of the variation and the specific base of the variation, e.g., 29903:A- > T.

**virus_id/genome_id:** The ID of the genome in which the variant occurs.

**taxon_id:** The mutated species.

**position:** Location of the variant on the chromosome.

**ref:** Reference nucleotides on the genome.

**alt:** The nucleotides that differ from the reference.

**type:** Variation type: single nucleotide variant or insertion/deletion.

**region:** An area of region variation that contains exonic, downstream and upstream.

**gene:** The gene corresponding to the variation.

**var_aa:** Amino acids before and after variant, e.g., D-G.

**var_aa_pos:** The relative position of the variant amino acid.

**synonymous:** Variation type: synonymous or nonsynonymous variant.

**mutation_frequency:** The frequency of variation.

**protein:** The corresponding protein in which the variant site occurs.

**var_aa_id:** The ID of occurrence variant sites, consisting of a **var_aa** field versus a **var_aa_pos** field, e.g., D614G.

**domain_id:** The ID of the domain to which the variant belongs, comprising the UniProt ID and the location of the domain, e.g., P0DTC2:13–685.

**domain_type:** The type of domain.

**description:** UniProt’s description of the domain feature.

Unique fieldss in case_genome.tsv:

**virus_id:** The id of the genome that matches.

**case_id:** The ID of the case that matches.

**curated:** Whether the matching results have been manually curated. TRUE indicates manually corrected and FALSE indicates scripted.

## Technical Validation

**Fig. 3:**
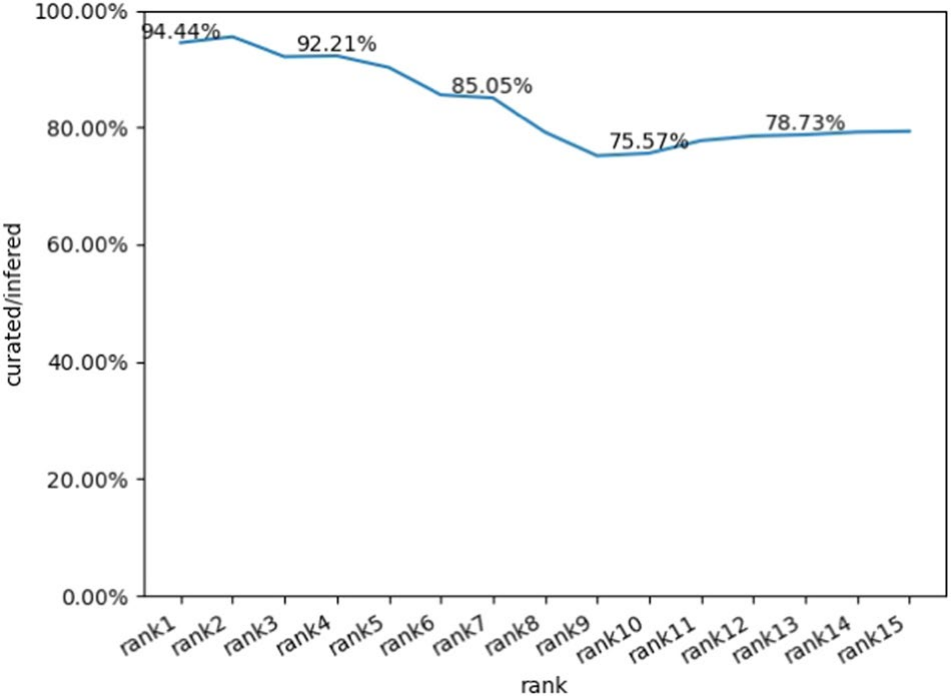
Top 1–15 rank Code matching accuracy.

Top 15 rank data in all inferred dataset was manual checked, and the accuracy was evaluated by different thresholds of top N rank. As shown in Fig. 3, the ranking increases, while the accuracy rate decreases, the accuracy of the data matched by the scripts was about 79% when the rank threshold decreased to 15.

As December 2019 to November 2020, we collected 445,470 genomic records and 2,571,621 epidemiological records, including 130 from manual curators, 3,684 from Wikipedia and 2,567,807 from Xu *et al*.’s data set. A total of 11,412 genome–case pairs were generated, 498 genome–case pairs from manually curate, 10,914 from code match. The 11,412 pairs, covers 1,178 genomes, and covers 9,942 cases, including 15 from manual curators, 234 from Wikipedia, and 11,163 from Xu *et al*.’s data set.

**Fig. 4:**
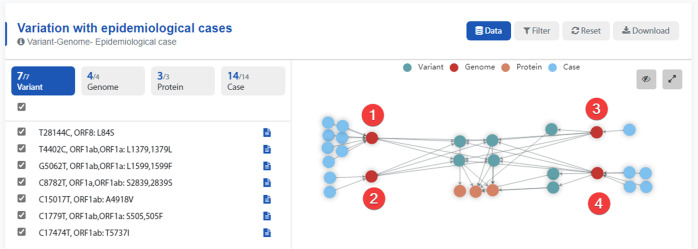
Variation in epidemiological cases. The green nodes in the figure represent the variation information, the red nodes represent the genomic information, the orange nodes represent the SARS-CoV-2 protein information, and the blue nodes represent the case information.

Figure 4 shows the earliest case matching data for South Korea. We found that the matching epidemiological data came from the same article^26^. Our cases 1–4 correspond to #3, #6, #11, and #21, respectively, in the article. Through the aforementioned article, we found the spread of case 1 to case 2 is also the first human–human transmission case found in Korea, and case 2 transmitted the infection to cases 3 and 4. Analysis of the variations of the genomes revealed that there are four common variation points in the genomes of the four cases: 4402:T-C(L1379L), 5062:G-T(G1167V), 8782:C-T(S2839S), and 28144:T-C(L84S). In cases 1 and 2, the genome variation is exactly the same, with only four variants listed. Case 3 has a unique variation: 17474:C-T(T5737I) and case 4 has two unique variations: 15017:C-T(A4918V) and 1779:C-T(F3838F). Using the graph visualization feature of the web application, the genomic mutation sites of SARS-CoV-2, the matched epidemiological information, and their connections can be intuitively visualized.

## Usage Notes

In the context of the COVID-19 pandemic, open sharing of data will help the world manage it. The user can through our web platform to access and use our data by https://www.biosino.org/kgcov/.

Most of the datasets available to date contain only epidemiological data^16,17^, without linking these data to the genome. Matching epidemiologic information with genomic information is beneficial for the surveillance of virus transmission and reconstruction of infection paths. Furthermore, these data provide supporting evidence for the molecular evolution of SARS-CoV-2 from another perspective^27–30^. However, it needs to be acknowledged that few detailed case reports have appeared in the later stages of the pandemic, creating some difficulty in the collection and matching of our epidemiologic data and resulting in a paucity of data for matching. We have tried our best to ensure the accuracy of the data. Please contact us promptly if you encounter any errors during its use.

## Data Availability

The bioinformatics tools used by the dataset are described under the Methods section. The parameters are as follows. BLAT(v. 36 × 5): -out = blast8 reference.fasta input.fasta out.blast8. MAFFT(v 7.453): --thread −16 --quiet in.fasta > out.ma. ANNOVAR(2019 Oct 24 version): convert2annovar.pl -format vcf4 -allsample -withfreq input.vcf > output.avinput, annotate_variation.pl -geneanno -dbtype refGene -buildver NC_045512v2 -out output.avoutput input.avinput annovar_ref/. The code used for data analysis are available on GitHub https://github.com/BioMedBigDataCenter/KGCoV/.
